# The Heterogeneity of Infiltrating Macrophages in Metastatic Osteosarcoma and Its Correlation with Immunotherapy

**DOI:** 10.1155/2021/4836292

**Published:** 2021-07-21

**Authors:** Zhanchao Wang, Huiqiao Wu, Yu Chen, Huajiang Chen, Wen Yuan, Xinwei Wang

**Affiliations:** Department of Orthopaedics, Changzheng Hospital, Naval Medical University, Shanghai 200003, China

## Abstract

**Background:**

Metastatic osteosarcoma is a common and fatal bone tumor. Several studies have found that tumor-infiltrating immune cells play pivotal roles in the progression of metastatic osteosarcoma. However, the heterogeneity of infiltrating immune cells across metastatic and primary osteosarcoma remains unclear.

**Methods:**

Immune infiltration analysis was carried out via the “ESTIMATE” and “xCell” algorithms in primary and metastatic osteosarcoma. Then, we evaluated the prognostic value of infiltrating immune cells in 85 osteosarcomas through the Kaplan–Meier (K-M) and receiver operating characteristic (ROC) curve. Infiltrations of macrophage M1 and M2 were evaluated in metastatic osteosarcoma, as well as their correlation with immune checkpoints. Macrophage-related prognostic genes were identified through Weighted Gene Coexpression Network Analysis (WGCNA), Lasso analysis, and Random Forest algorithm. Finally, a macrophage-related risk model had been constructed and validated.

**Results:**

Macrophages, especially the macrophage M1, sparingly infiltrated in metastatic compared with the primary osteosarcoma and predicted the worse overall survival (OS) and disease-free survival (DFS). Macrophage M1 was positively correlated with immune checkpoints PDCD1, CD274 (PD-L1), PDCD1LG2, CTLA4, and TIGIT. In addition, four macrophage-related prognostic genes (IL10, VAV1, CD14, and CCL2) had been identified, and the macrophage-related risk model had been validated to be reliable for evaluating prognosis in osteosarcoma. Simultaneously, the risk score showed a strong correlation with several immune checkpoints.

**Conclusion:**

Macrophages potentially contribute to the regulation of osteosarcoma metastasis. It can be used as a candidate marker for metastatic osteosarcoma' prognosis and immune checkpoints blockades (ICBs) therapy. We constructed a macrophage-related risk model through machine-learning, which might help us evaluate patients' prognosis and response to ICBs therapy.

## 1. Introduction

Osteosarcoma is a common malignant bone tumor, and its event-free survival rate is 59% at three years and 54% at five years [1]. Osteosarcoma mainly occurs in long bones of children and adolescents, with 15–20% of patients initially diagnosed with clinically detectable metastases, and pulmonary metastasis is the most adverse factor at diagnosis [1, 2]. Neoadjuvant chemotherapy combined with surgical resection is a major therapeutic strategy for metastatic osteosarcoma [2]. However, increased chemotherapy toxicity failed to improve patients' survival outcomes [3]. Apart from metastasis, there is no other prognostic factor that can stratify patients [2].

Accumulating evidence report that the tumor-infiltrating immune cells play a regulatory role in the nourishment, maintenance, proliferation, and metastasis of osteosarcoma, thereby affecting the patients' survival [4, 5]. But a few studies focused on metastatic osteosarcoma. Infiltrating immune cells consist of T lymphocytes and B lymphocytes along with myeloid cells like tumor-associated macrophages, dendritic cells, and myeloid-derived suppressor cells [6]. The evaluation of the specific density of infiltrating immune cells not only has clinical significance but also guides the immune checkpoints blockades (ICBs) therapy [7, 8]. There is controversy about the correlation between infiltrating immune cells and osteosarcoma prognosis in different studies. Several reports have indicated that infiltrating immune cells were positively correlated with better clinical survival [9, 10]. While Chen et al. and Koirala et al. have reported the opposite conclusion that the infiltrating immune cells act as a risk factor for osteosarcoma [11, 12], M1 and M2 phenotypes were two polarized terminals of the macrophages that exhibit opposite functions in antitumor response [13]. Macrophage M1 contributes to tumor elimination and M2 serves as a promoter in tumorigenesis. Contrary to the cancer-promoting role of macrophages in a wide range of cancers, the high density of macrophages indicates longer survival in osteosarcoma [13]. However, there is a lack of systematic analysis of the heterogeneity of infiltrating immune cells in metastatic osteosarcoma.

**Figure 1:**
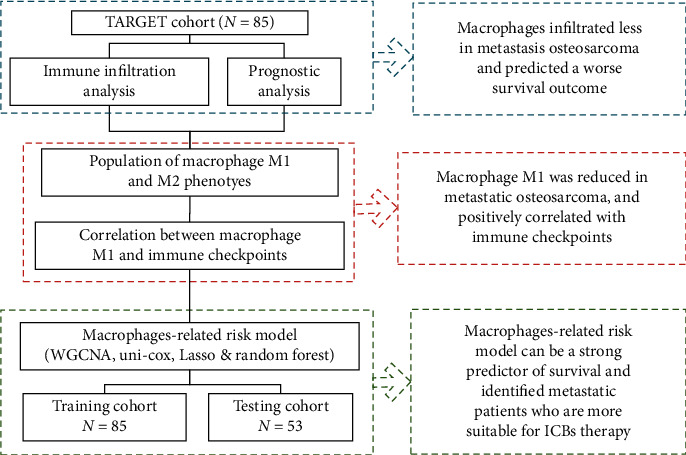
Workflow of this study.

The workflow of the current study is shown in Figure 1. We comprehensively analyzed the population of infiltrating immune cells in metastatic compared with the primary osteosarcoma and their clinical significance. We observed metastatic tissues were less infiltrated with macrophages, especially the M1 phenotype. And macrophage M1 was positively correlated with the expression of immune checkpoints. A macrophage-related risk model had been developed through WGCNA and machine-learning tools, which could be a strong predictor of survival and identification of osteosarcoma patients who are more suitable for ICBs therapy.

## 2. Methods

### 2.1. Data

The high throughput sequencing FPKM data of osteosarcoma tissues with corresponding clinical information (gender, race, age, metastatic state, overall survival, and disease-free survival) were retrieved and downloaded from the Therapeutically Applicable Research to Generate Effective Treatments (TARGET, https://ocg.cancer.gov/programs/target) program. 85 osteosarcoma patients (21 metastatic and 64 primary tissues) with a follow-up period of more than 30 days were included in this study. Series GSE21257 (53 osteosarcoma patients, GPL10295) from Gene Expression Omnibus (GEO, https://www.ncbi.nlm.nih.gov/geo/) was selected for external validation of macrophage-related risk model [14].

### 2.2. Immune Infiltration Analysis

“ESTIMATE” (Estimation of STromal and Immune cells in MAlignant Tumor tissues using Expression data) is a method that uses specific gene signature to calculate the fraction of stromal/immune cells in solid tumor [15]. In this study, *R* package “estimate” (with default parameters) was performed in the TARGET cohort to quantify tumor purity and the infiltrations of stromal/immune cells in osteosarcoma tissues [15]. Furthermore, the “xCell” (R package) algorithm was also carried to infer the populations of infiltrating immune cells in osteosarcoma [16].

### 2.3. Identification of Macrophage-Related Gene Coexpression Modules by Weighted Gene Coexpression Network Analysis (WGCNA)

To identify the macrophage-related gene coexpression gene clusters. Weighted Gene Coexpression Network Analysis (WGCNA, *R* package “WGCNA”) was performed on the immune genes acquired from ImmPort Portal (https://www.immport.org/home) and immune parameters (immune score, macrophages, and macrophage M1) [17]. The optimal soft threshold (power) was found to be three. And the similarity matrix was next converted into a weighted adjacent matrix. Furthermore, both the topology overlay metric (TOM) and DynamicTreeCut (a bottom-up algorithm) were used to identify weighted gene coexpressed modules. Module eigengenes (MEs) were defined as the first principal component of each module and represent the gene expression of the modules. The relationship between modules/MEs and immune parameters (immune score, infiltration of macrophages, and macrophage M1) was assessed by Pearson correlation analysis. Modules with *p* < 0.05 seemed as macrophage-related modules for subsequent analyses.

### 2.4. Gene Ontology (GO) Analysis

Gene Ontology (GO) analysis was performed for genes in a significant module by using *R* package “clusterProfiler” to identify related biological processes (BP) [18]. The adj *p* value < 0.05 was considered statistically significant.

### 2.5. Protein-Protein Interaction (PPI) Network

The PPI network was summarized by using the STRING database (Version 11.0, https://www.string-db.org/) and visualized via Cytoscape (Version 3.7.0). The interconnectivity degree of each node within the network was calculated. Molecular Complex Detection (MCODE) with default parameters was then used to acquire densely connected clusters.

### 2.6. Construction of Macrophage-Related Risk Model via Machine-Learning

In the significant module, genes with the *p* < 0.05 in univariate cox (unicox) analysis were considered as prognostic genes [19], followed by machine-learning Lasso-penalized cox analysis (tenfold cross-validation) and Random Forest algorithm to reduce the number of prognostic genes [20, 21]. Four macrophage-related prognostic genes IL10, VAV1, CD14, and CCL2 were identified, and a macrophage-related risk stratification model was constructed based on multivariate cox (multicox) regression with the expression of these genes. Risk score = IL10 *∗* (−0.633) + VAV1 *∗* (−0.5541) + CD14 *∗* (−0.2482) + CCL2 *∗* (−0.4807). The osteosarcoma patients were classified into two subsets based on the median risk scores; if risk score > median risk scores, classify into the high-risk group; if risk score < median risk scores, classify into the low-risk group.

### 2.7. Statistics

All statistical analyses were performed in *R* (Version 3.4.1) and its appropriate packages. Continuous variables were analyzed using Student's *t*-test or Wilcox test. Correlation analysis was based on the Spearman method. Overall survival (OS) and disease-free survival (DFS) were used as the survival outcomes and were estimated by the Kaplan–Meier (*K*-*M*) analysis, which was based on a log-rank test. And receiver operating characteristic (ROC) curves were used to evaluate the performance of the prognostic factors. *p* < 0.05 was seen as statistically significant.

## 3. Results

### 3.1. The Heterogeneity of Infiltrating Macrophages and Its Clinical Significance in Metastatic Osteosarcoma

**Figure 2:**
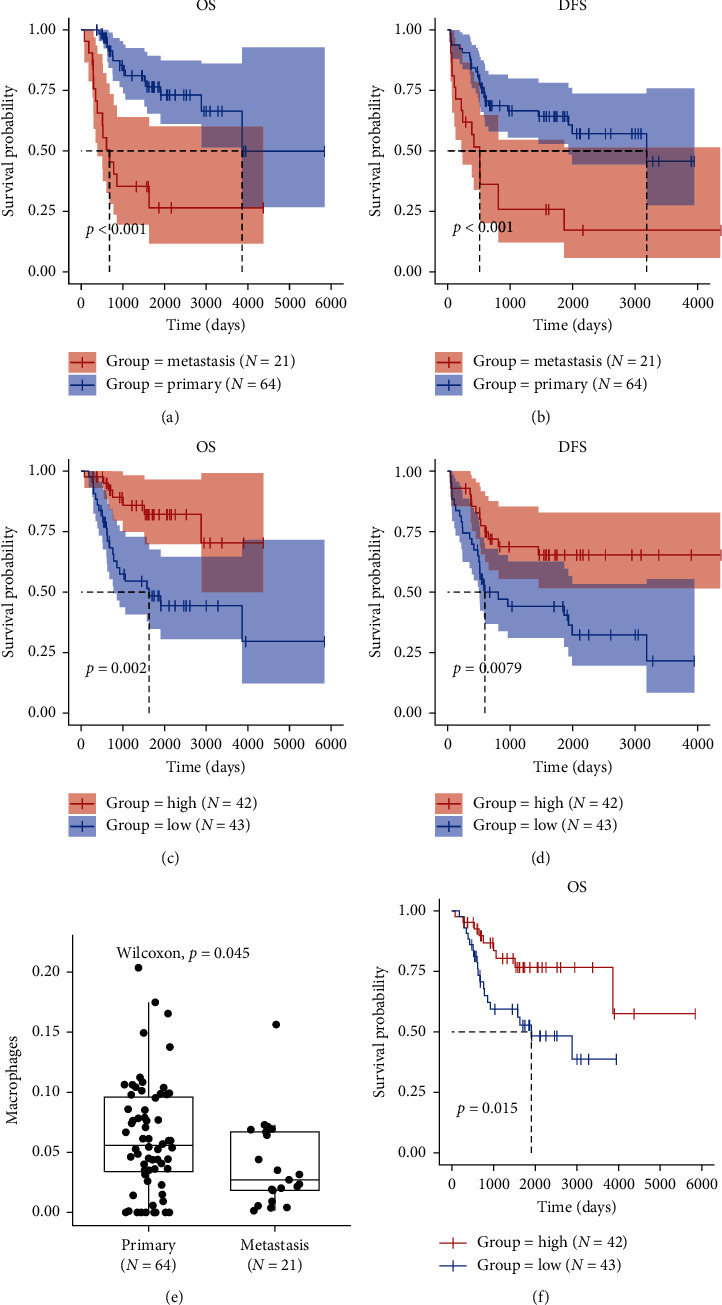
The heterogeneity of infiltrating macrophages and its clinical significance in metastatic osteosarcoma. (a-b) Association of the metastatic state with (a) OS and (b) DFS; the patients were divided into two groups according to the metastatic state, as shown in the *K*-*M* curves; the survival outcomes in metastatic osteosarcoma were worse. (c-d) Osteosarcoma patients were classified to high (red line) and low groups (blue line) based on the median value of immune score. *K*-*M* curves show that the patients with lower immune score had (c) greater mortality and (d) shorter DFS than does high immune score. (e) Distribution of infiltrating macrophages in primary and metastatic osteosarcoma. Box-plot shows that macrophages infiltrated less in metastatic osteosarcoma (*p*=0.05). (f) *K*-*M* analysis shows that patients with high infiltrating macrophages (red line) have longer OS. Abbreviation: OS : overall survival; DFS : disease free survival; *K*-*M* : Kaplan–Meier.

The Kaplan–Meier (*K*-*M*) analysis was performed to describe the effect of metastatic state on osteosarcoma survival outcomes. And the discrepancy of survival outcomes across primary (*N* = 21) and metastatic osteosarcoma patients (*N* = 64) had been observed; patients with metastasis had decreased overall survival (OS) and disease-free survival (DFS) (Figures 2(a) and 2(b), all *p* < 0.001). The “ESTIMATE” algorithm was then applied to gain insight into the heterogeneity of infiltrating immune cells between primary and metastatic osteosarcoma. But no statistical differences were found in a comparison of stromal score/immune score between them, although stromal/immune cells tended to infiltrate less in metastatic osteosarcoma (Supplementary Figure 1). Nevertheless, we still evaluated the prognostic value of immune score in osteosarcoma. The patients were categorized into high (*N* = 42) and low groups (*N* = 43) according to the median immune score. Patients with high immune score had remarkably longer OS (Figure 2(c), *p*=0.002) and DFS (Figure 2(d), *p*=0.008). The high predictive ability of immune score was confirmed by the receiver operating characteristic (ROC) curve (Supplementary Figure 2).

Infiltrating immune cells included the lymphocytes like CD4^+^/CD8^+^ T cells, B cells, and natural killer (NK) cells, monocytes and dendritic cells [22]. Herein, we mapped the population of infiltrating immune cells using the “xCell” algorithm. And the decreasing macrophages were observed in metastatic compared with primary osteosarcoma (Figure 2(e), *p*=0.045). Additionally, patients with lower macrophages exhibited significantly shorter OS (Figure 2(f), *p*=0.015). As for the metastatic site, the infiltration of macrophages was not a significant difference between patients with different metastatic sites (only lung versus bone and lung, Supplementary Figure 3). We concluded that macrophages poorly infiltrated in metastatic osteosarcoma with high prognostic value.

### 3.2. Dysregulation of Infiltrating Macrophage M1 and Its Correlation with Immune Checkpoints

**Figure 3:**
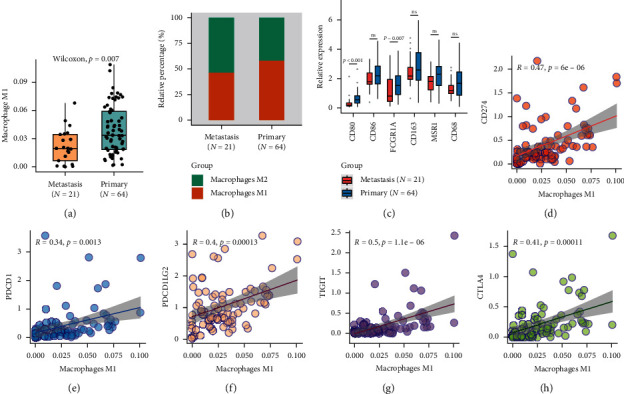
Dysregulation of infiltrating macrophage M1 and its correlation with immune checkpoints. Box-plots show that the absolute amount (a) and the relative proportion (b) of macrophage M1 were reduced in metastatic osteosarcoma. (c) The expression of the macrophage M1 biomarkers CD80, CD86, FCGR1A and M2 biomarkers CD163, MSR1, CD68 in primary (orange box) and metastatic osteosarcoma (blue box). Box-plot shows that the M1 phenotype biomarkers were downregulated in metastatic osteosarcoma. Correlation of macrophage M1 and immune checkpoints, (d) CD274, (e) PDCD1, (f) PDCD1LG2, (g) TIGIT, (h) CTLA4. *R* correlation coefficient.

Fully polarized macrophages (M1 and M2 phenotypes) have extremely divided functions in antitumor response, tumorigenesis inhibitor macrophage M1 (CD80^+^, CD86^+^, and FCGR1A^+^) and promoter macrophages M2 (CD163^+^, MSR1 ^+^, and CD68 ^+^) [23–25]. The dynamic of M1/M2 polarization plays an important role in tumorigenesis, removal, and restoration of abnormal macrophages which has been proposed as one potential therapy for patients with tumors [26]. In this study, both the absolute amount and the relative proportion of macrophage M1 were significantly reduced in metastatic tissues (Figure 3(a), *p*=0.007). The expression of M1 phenotype markers CD80 and FCGR1A were also downregulated in metastatic osteosarcoma (Figure 3(b)), suggesting that the decreased macrophage M1 might associate with the osteosarcoma metastasis.

ICBs therapy has drawn great success in immunotherapy, and the immune checkpoints are emerging predictive biomarkers for ICBs response. This study found that macrophage M1 was positively correlated with immune checkpoints PDCD1, CD274 (PD-L1), PDCD1LG2, CTLA4, and TIGIT (Figure 3(c)), suggesting the opportunity of ICBs therapy in osteosarcoma patients with high infiltrating macrophage M1.

### 3.3. Identification of Macrophage-Related Modules by Weighted Gene Coexpression Network Analysis

**Figure 4:**
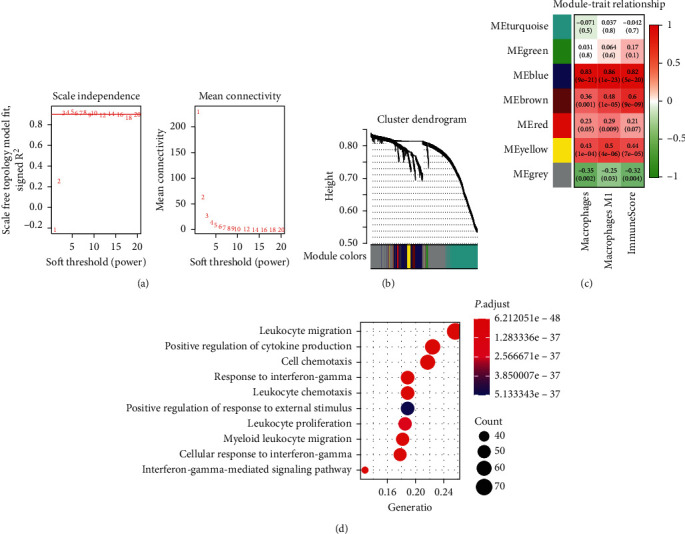
Identification of macrophage-related modules by Weighted Gene Coexpression Network Analysis. (a) Analysis of the scale free network for various soft thresholding powers. (b) Clustering dendrograms and modules identified by WGCNA. As shown in the result, six gene coexpression modules are constructed and are shown with different colors. The list of genes within the six modules is listed in Supplementary Table 2. (c) Heatmap of the correlation between modules and immune phenotypes (immune score, infiltration of macrophages, and macrophage M1). The correlation coefficient (up) and *p* value (down) are annotated in each cell. (d) Biological process of GO analysis for genes within the blue coexpression module, top 10 GO categories with adj. *p* < 0.05. Abbreviation: WGCNA : Weighted Gene Coexpression Network Analysis; GO : Gene Oncology.

To identify the macrophage-related gene coexpression modules, WGCNA was applied based on the expression profile of 1709 immune-related genes (Supplementary Table 1). Firstly, the hierarchical clustering analysis was performed to eliminate outlier samples and ultimately left 79 samples (Supplementary Figure 4). Then, three were selected as the optimal soft threshold (power) for this study (Figure 4(a)). A total of six modules were identified and the gray module contained genes that were not assigned to any module (Figure 4(b)). The genes in each module were listed in Supplementary Table 2. This study conducted correlation analysis of immune phenotypes (immune score, infiltration of macrophages, and macrophage M1) and gene coexpression modules. The result showed that the blue module was strongly correlated with immune score (Cor = 0.83, *p* < 0.001), macrophages (Cor = 0.86, *p* < 0.001), and macrophage M1(Cor = 0.82, *p* < 0.001) (Figure 4(c)).

Immune-related GO categories were enriched in the blue module, including leukocyte migration (GO:0050900), positive regulation of cytokine production (GO:0001819), response to interferon-gamma (GO:0034341), positive regulation of response to external stimulus (GO:0032103), leukocyte proliferation (GO:0070661), myeloid leukocyte migration (GO:0097529), and interferon-gamma-mediated signaling pathway (GO:0060333) (Figure 4(d)). More enriched GO terms are summarized in Supplementary Table 3.

**Figure 5:**
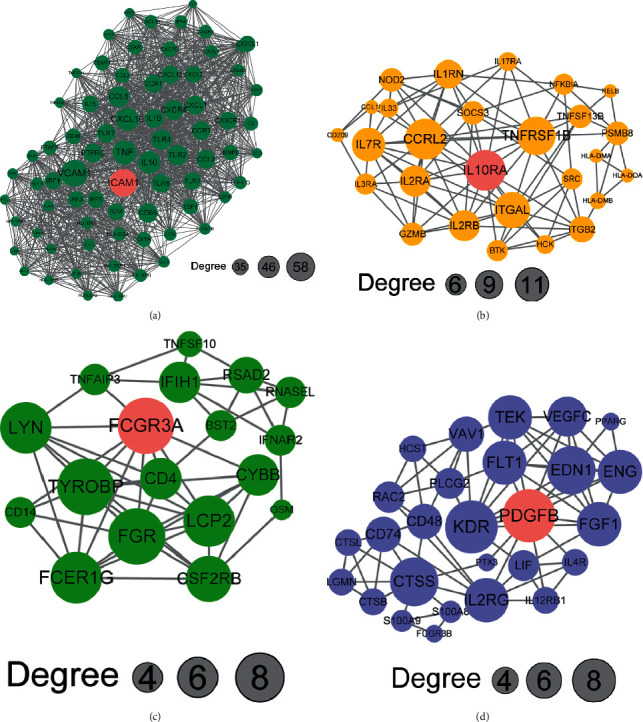
Protein-protein interaction network of densely connected clusters in blue module. (a-d) The size of a node in the PPI network indicates the interconnection degree. Abbreviation: PPI : protein-protein interaction.

Through STRING database and Cytoscape, we retrieved and reconstructed the PPI network of the blue module, which contains 273 nodes and 4906 edges. Four key functional clusters were extracted using MCODE in Cytoscape. For convenience, we named them ICAM1, IL10RA, FCGR3A, and PDGFB, respectively (Figures 5(a)–5(d)). In the ICAM1 module (Figure 5(a)), 1465 edges are formed in the network which involves 75 nodes, of which ICAM1, VCAM1, TNF, CXCL10, and IL10 were significant nodes because of the high interconnection degree. CXCL10 was predominantly expressed by macrophages and was associated with ICBs response [27]. Highly interconnected nodes in four clusters, including IL10RA, CCRL2, and PDGFB were related to the polarization, accumulation of macrophages [28–30].

### 3.4. Construction and Validation of the Macrophage-Related Risk Model

**Figure 6:**
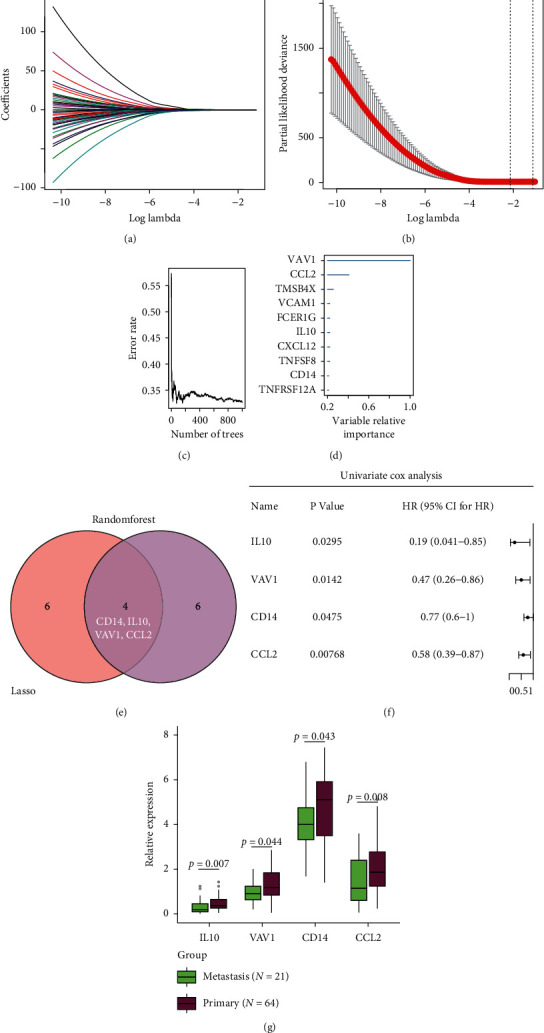
Identification of macrophage-related prognostic genes. (a) Lasso coefficient profiles of 49 potential prognostic genes. (b) Lasso regression with tenfold cross-validation obtains ten prognostic genes using minimum lambda value. (c) Left panel shows the relationship between variables and accuracy in RFE-RF predictor, and (d)right panel shows the variable relative importance. (e) Venn diagram shows the overlap between Lasso and Random forest. (f) The result of unicox shows that all four macrophage-related prognostic genes are protective genes with HR < 1. (g) The distribution of four macrophage-related prognostic genes IL10, VAV1, CD14, and CCL2 in metastatic and primary tissues. Abbreviation: RFE-RF : Recursive Feature Elimination-Random Forest; unicox: univariate cox; HR : hazard ratio.

**Figure 7:**
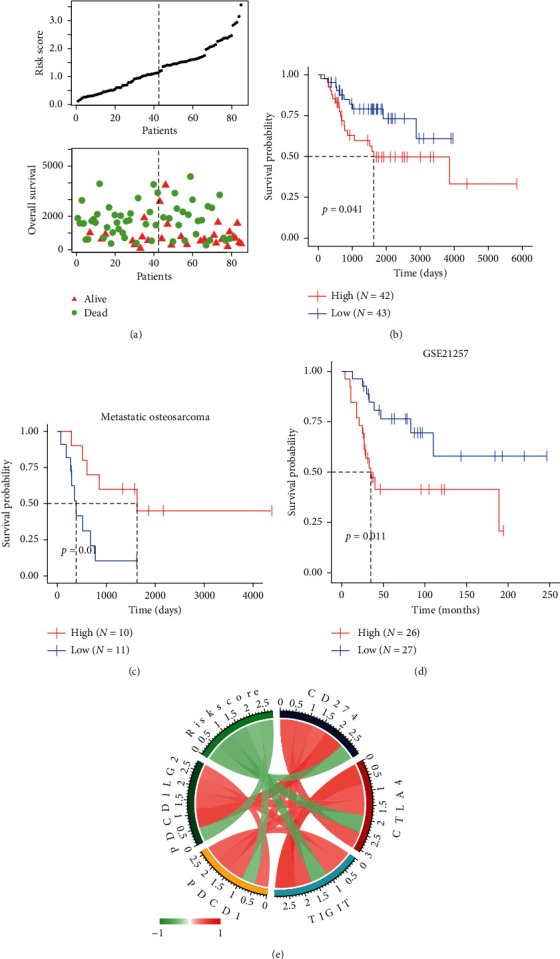
Construction and validation of the macrophage-related risk model. (a) Up panel shows the risk score of the high- and low groups, while the down panel shows the overall survival of osteosarcoma patients. (b) The prognostic value of the risk model in 85 osteosarcoma patients of the entire TARGET database. (c) The prognostic value of the risk model in metastatic osteosarcoma. (d) Validation of the prognostic value of the risk model in GSE21257. (e) Correlation between risk score and immune checkpoints.

Unicox analysis was applied on genes in the significant module (blue module); 49 out of 105 genes were screened as potential prognostic genes (Supplementary Table 4). Lasso analysis and Random Forest algorithm were performed to identify four prognostic genes, IL10, VAV1, CD14, and CCL2 (Figures 6(a)–6(d)). All of them were protective genes with HR < 1 (Figure 6(e)), as well as being downregulated in metastatic tissues (Figure 6(f)). The macrophage-related risk model based on these genes was as follows: risk score = IL10 *∗* (−0.633) + VAV1 *∗* (−0.5541) + CD14 *∗* (−0.2482) + CCL2 *∗* (−0.4807). The distribution of risk score is shown in Figure 7(a), the risk model can efficiently stratify the osteosarcoma patients with distinct OS both in the entire training cohort and subcohort (metastatic patients, *N* = 21, Figures 7(b) and 7(c)). The risk score was negatively correlated with the expression of immune checkpoints (Figure 7(e), Supplementary Table 5). Furthermore, the accuracy of the risk model was validated in external cohort GSE21257 (Figure 7(d)).

## 4. Discussion

The interplay of infiltrating immune cells and the cancer cell critically affects osteosarcoma metastasis [31]. However, few previous studies have characterized the landscape of infiltrating immune cells in metastatic osteosarcoma. In the current work, we attempted to systematically analyze the characteristics of infiltrating immune cells and their clinical significance in metastatic osteosarcoma using several bioinformatics tools. Immune infiltration analysis showed that the abundance of macrophages was decreased in metastatic than in primary tissues, which indicated poor survival outcomes, suggesting that macrophages may alleviate the metastasis. These results are consistent with a previous clinical trial that macrophages can be used as an independent prognostic biomarker in osteosarcoma [32]. The dynamic balance between macrophage M1 (tumor inhibitor) and M2 (tumor promoter) can exert an influence on antitumor response and tumor's fate [33]. Mahlbacher et al. highlighted that the antitumor effect of macrophage M1 is pronounced [34]. We observed that both absolute quantitative and proportion of macrophage M1 were reduced in metastatic osteosarcoma, referring to a protumor phenotype.

ICBs have shown safety, well tolerability, and clinical benefit in a variety of cancers [35]. And the expression of immune checkpoints is an effective predictive biomarker for ICBs therapy [36]. Unfortunately, TARGET datasets from the online public databases did not contain information about ICBs therapy. Thus, correlations between infiltrating immune cells and immune checkpoints were conducted in this study to refer to the efficacy of ICBs. We found that macrophage M1 was positively correlated to PDCD1, CD274 (PD-L1), PDCD1LG2, CTLA4, and TIGIT. Considering the low abundance of macrophage M1 in metastatic osteosarcoma, we speculated that metastatic patients may benefit less from ICBs therapy compared with the primary. And more clinical trials are needed to confirm this conclusion. Patients with low infiltrating macrophage M1 are more likely to benefit from the combination of cytokine administration and ICBs therapy [37]. Detailly, granulocyte macrophage colony-stimulating factor (GM-CSF) can promote macrophages' influx and M1-like polarization, thereby improving the efficiency of ICBs therapy [38, 39].

The traditional prognostic evaluation for osteosarcoma mainly relies on the metastatic state [1, 40]. Its predictive effect is far from satisfactory due to the molecular and genetic heterogeneity. Immune-based prognostic systems rekindle hope for improving the clinical outcomes of osteosarcoma over that of the single clinical factor. In this context, we identified four macrophage-related prognostic genes, IL-10, VAV1, CD14, and CCL2, via combining the WGCNA, PPI, and machine-learning. All of them were protective genes in this study, and the roles of VAV1 in osteosarcoma still have not been reported.

Interleukin 10 (IL-10), an anti-inflammatory cytokine, is produced primarily by macrophages and neutrophils [41]. IL10 may elicit its antitumor effects through multiple mechanisms, high expressed IL-10 is associated with improved DFS, and the efficiency of a combination of Pegilodecakin (Pegylated IL-10) and ICB was compelling [42, 43]. However, Kaplanov et al. verified that macrophages-derived IL-10 is dominant in the tumor microenvironment and leads to immunosuppression and progression of breast cancer [44]. These inconsistent results may be due to the fact that IL-10 involved pathway is dynamic and complex in the tumor microenvironment, and it can be a double-edged sword in tumor progression. VAV1 (Vav guanine nucleotide exchange factor (1) is a guanine nucleotide exchange factor (GEF); its expression can be triggered by IL-10 [45]. A recent study showed that VAV1 acts as a tumor suppressor in leukemia by promoting the degradation of ICN1 (intracellular domain of Notch1), while VAV1 mutation facilitates the malignant transformation of T cells [46, 47]. VAV1 inhibits the metastasis and therapy resistance by downregulating the Akt2 (AKT serine/threonine kinase 2) signaling pathway in pancreatic cancer [48]. CD14 is a surface antigen that is preferentially expressed on macrophages and plays a central role in macrophage M2 polarization [49]. CD14+ macrophage M2 is associated with reduced metastasis and better survival in osteosarcoma [14]. But overexpression of CD14 correlates with increased malignancy in breast cancer and colorectal cancer [50, 51]. CCL2 (C–C motif chemokine ligand 2) is overexpressed in liver cancer; it can drive the recruitment of macrophages [52, 53]. Sanford et al. conducted a clinical study and found that the CCL2 is a prognostic factor, which is related to the decreased survival in pancreatic cancer [54]. Meanwhile, the blockade of the CCL2 signaling pathway resulted in decreased recruitment of inflammatory monocytes and macrophages in the tumor microenvironment, forming an immunosuppressive phenotype and conferred cancer cells proliferation [53].

Herein, we established a macrophage-related risk model based on the expression of IL10, VAV1, CD14, and CCL2. This could overcome the prognostic limitations in these patients with the same metastasis state. In addition, this prognostic model could be effectively used to assess the survival outcomes in both osteosarcoma and metastatic osteosarcoma, meaning that the models can be used as markers for the prognosis of osteosarcoma patients. Importantly, the risk score was positively correlated with the expression of immune checkpoints, which provide an excellent opportunity for ICBs therapy for metastatic patients with a high-risk score.

## 5. Conclusion

In summary, this study indicated heterogeneity of infiltrating macrophages in primary and metastatic osteosarcoma. A novel macrophage-related risk model was constructed and validated, which could be a strong predictor of survival and identified metastatic patients who are more suitable for ICBs therapy. However, the immune phenotype does not only depend on the single macrophages; the potential association of macrophages with metastasis in osteosarcoma needs to be further studied in the future.

## Data Availability

The TARGET cohort and GSE21257 used in this study are available from the corresponding links: TARGET cohort: https://ocg.cancer.gov/programs/target; GSE21257: https://www.ncbi.nlm.nih.gov/geo/query/acc.cgi?acc=GSE21257.

## Supplementary Materials

Supplementary Materials Supplementary Figure 1: the stromal score and immune score between primary and metastatic osteosarcoma in TARGET database. Supplementary Figure 2: time-dependent ROC analysis indicating higher predictive value of immune score in OS (left panel, 3 years AUC = 0.702, 5 years AUC = 0.702) and DFS (right panel, 3 years AUC = 0.654, 5 years AUC = 0.644). Abbreviation: ROC : receiver operating characteristic curve. Supplementary Figure 3: the box plot shows the macrophages' infiltration between patients with different metastatic sites (only lung versus bone and lung). Supplementary Figure 4: sample clustering to detect outliers; 85 osteosarcoma patients were in the clusters and 79 patients have passed the cut.
