# Supplementary material for: The Heterogeneity of Infiltrating Macrophages in Metastatic Osteosarcoma and Its Correlation with Immunotherapy
Source: J Oncol. 2021 Jul 21;2021:4836292. doi: 10.1155/2021/4836292 (PMC8321719; doi:10.1155/2021/4836292)
Supplement: Supplementary Materials — Supplementary Figure 1: the stromal score and immune score between primary and metastatic osteosarcoma in TARGET database. Supplementary Figure 2: time-dependent ROC analysis indicating higher predictive value of immune score in OS (left panel, 3 years AUC = 0.702, 5 years AUC = 0.702) and DFS (right panel, 3 years AUC = 0.654, 5 years AUC = 0.644). Abbreviation: ROC : receiver operating characteristic curve. Supplementary Figure 3: the box plot shows the macrophages' infiltration between patients with different metastatic sites (only lung versus bone and lung). Supplementary Figure 4: sample clustering to detect outliers; 85 osteosarcoma patients were in the clusters and 79 patients have passed the cut. [file 4836292.f1.zip › 4836292.f1/Supplementary Tables.docx]

**Supplementary Table**

**Supplementary Table 1:** List of 1709 immune related genes from ImmPort database

**Supplementary Table 2:** List of genes in each gene co-expression module from WGCNA

**Supplementary Table 3:** Summary of top 20 GO terms (Biological process) that genes within the blue module enriched

**Supplementary Table 4:** Result of univariate cox analysis

**Supplementary Table 5:** The correlation between risk score and immune checkpoints

**Supplementary Table 1: List of 1709 immune related genes from ImmPort database**

| *FGR, NFYA, SEMA3F, PLXND1, CALCR, MPO, ITGAL, CRLF1, MAP3K14, TAC1, CX3CL1, TNFRSF12A, CCL26, ADIPOR2, NOS2, CD79B, NOX1, PGLYRP1, IL32, MASP2, GIPR, SEMA3G, CD4, BTK, HFE, FYN, PLAUR, TYROBP, CD22, SEMA3B, LTF, NR1H4, PSMC4, BID, IL20RA, IGF1, SLC11A1, MARCO, CD74, HGF, NR1H3, TYMP, VIM, FAS, IFNGR1, TNFRSF1B, RABEP1, GRN, TMSB10, FLT4, MSR1, CDH1, TNC, TG, LCP2, ADRB1, HSPA5, TNFRSF17, KITLG, UTS2, TNFRSF9, LTBP1, ELN, PTGER3, PIK3CB, KCNH2, EIF2AK2, IL17RB, OGFR, NGFR, RFXANK, CALCRL, GLP2R, PRKCQ, ELAVL1, TIE1, NFYC, FGFR2, NEO1, TNFRSF1A, RHOA, FGFR3, BCL3, GAL, RORA, TGFBR3, NEDD4, SCT, FGF10, FGF22, CDC42, NCK2, PRLH, TFRC, FCGR2B, NFATC3, PTGS2, EED, NOX3, MYDGF, TXK, SEMA3A, SEMA3C, FGF4, PLXNA2, RARB, IL4R, PAK3, FGFR1, ARAF, EDN1, ADCYAP1R1, FGF20, BPIFB2, SCTR, HSP90AA1, CXCL2, ARG2, PTPRC, IL12RB2, PGR, OPRK1, SEMA5B, GSK3B, CYLD, TXLNA, NOX4, PSMC5, CETP, SH3BP2, PTHLH, DEFB127, MAVS, OAS1, CHGB, NOS1, CMTM1, BIRC5, LTBP4, ICAM1, LYZ, THPO, FLT3LG, CD209, IL5RA, CMTM6, APOH, ESR1, CMA1, PSME1, SLC22A17, SEMA6A, TGFB2, PSMD5, SEMA4G, BLNK, IL11, PGC, NCR2, HSP90AB1, MLN, JAK2, IL12RB1, NRP1, PSMD8, SERPIND1, OSM, MAPK1, DDX17, HMOX1, APOBEC3H, PDGFB, GRAP2, CSF2RB, IL2RB, CTSG, GZMB, SOS2, PSMC6, LGMN, CHGA, PSMC1, NFKBIA, PSME2, NFATC4, PLTP, MMP9, TRPC4AP, PROCR, CD40, R3HDML, HNF4A, NFATC2, BMP7, AVP, ANGPT4, PDYN, HCK, JAG1, OXT, BPI, CST4, WFDC2, EPPIN, PSMD10, TLR8, CD40LG, FGF14, TNFSF13B, MLNR, FGF9, FLT1, MAPK3, NFAT5, CCL22, CCL17, PSMD7, IL21R, AQP9, CSK, PDGFRL, IKBKB, NDRG1, IL7, TNFRSF10A, CGB2, NFKBIB, LHB, RELB, FCGRT, AMH, RETN, CLEC4M, IL27RA, GPI, AKT2, EBI3, TGFB1, CD79A, TYK2, CLEC11A, FGF21, PIK3R2, HAMP, NAMPT, PIK3CG, PTN, ZC3HAV1, MET, LMBR1, VIPR2, NOD1, CRHR2, GHRHR, CCL24, TFR2, TGFBR1, C5, OGN, TNFSF8, ENG, RLN2, RLN1, DDX58, PTGDS, CXCL12, MAPK8, PPP3CB, BMPR1A, FGF8, MAP3K8, DKK1, CSF3, PSMD3, ICAM2, PSMD11, CCL7, CCL2, CCL8, CCL1, DHX58, PPY, VTN, GNRHR, PF4V1, NFKB1, AREG, IL2, HSPA8, UNC93B1, IL10RA, CBL, MDK, CD81, CALCA, IL23A, FGF6, LTBR, ENDOU, VDR, IL26, IFNG, PTPN6, ULBP1, PPARD, OPRM1, MAPK14, IL17A, IL17F, GLP1R, BMP5, BACH2, NR2E1, CCR6, VEGFA, SEMA5A, GHR, BRD8, HBEGF, ITK, IL12B, NPR3, PRLR, IL4, IL5, FGF1, NR3C1, LIFR, PDGFRB, STC2, KNG1, HRG, CD86, RBP2, RBP1, WNT5A, FGF12, CBLB, PLXNA1, CSPG5, ACVR2B, VIPR1, IL1A, CCL20, ZAP70, POMC, PSMD14, GCG, IFIH1, TACR1, LANCL1, REG1A, STAT1, GNLY, IL1R2, IL1R1, IL1RL2, IL1RL1, IL18R1, IL18RAP, PROC, SDC1, SOS1, CACYBP, ANGPTL1, OPRD1, HDAC1, LEPR, NR5A2, BMP8B, AKT3, CD48, CR2, MPL, ARTN, PRDX1, PIK3R3, FASLG, TNFSF4, NENF, NRP2, CREB1, TNFAIP3, GHRH, SPP1, FGF23, LCN1P1, NR4A3, CSF3R, PGF, LTBP2, TGFB3, ESRRB, PRLHR, CRHR1, TEK, INSL6, INSL4, IFNA6, IFNA8, TNFSF18, TNFSF11, NR2C1, NFYB, TNFRSF10B, PTK2B, PPP3CC, NPPB, TNFRSF8, IAPP, CAT, FABP3, CCRL2, GHSR, TNFSF10, PIK3CA, CXCR4, ACVR2A, FLT3, PAEP, OBP2A, CD244, PTGFR, NPY, INHBA, ACO1, CNTFR, SFTPA1, PLAU, ECD, NR4A1, LRP1, IL13RA2, ACVR1C, INHA, MC3R, PI3, SLPI, SDC4, SEMG2, PLCG1, EDN3, SEMG1, IL9R, IL17C, NDP, CXCL6, EREG, AHNAK, SLC10A2, ABCC4, IRF1, AMELX, BMP4, PTGER2, KIR2DL1, OPRL1, IL1B, IL37, PSPN, TNFSF9, CD70, C3, TNFSF14, GNRH2, DEFB126, BMP2, PCSK2, DEFB129, GDF5, BPIFB1, SLURP1, HCST, THRA, CCR7, NR1D1, IRF3, SBDS, HTN1, BECN1, PRKCG, HSPA2, AVPR2, MAP2K2, CANX, EDN2, MASP1, IL22, FGFRL1, WFIKKN1, IL17B, TNFRSF19, GNAI1, KDR, ADM2, MCHR1, GALR3, RAC2, LIF, APOBEC3A, APOBEC3F, VGF, IRF5, DLL4, ACKR4, FGF13, SHC2, INS-IGF2, LBP, GDF1, BST2, BMP15, EPO, GDF15, JUND, PAK4, ADRM1, GMFG, ANGPTL6, PLXNA3, ULBP2, ULBP3, BPIFA2, BPIFA3, DEFB118, GFAP, PYY, CCL25, NFATC1, IDO1, TRAF3, NR1H2, PSME3, IL13RA1, RARA, FSHB, NR0B2, RAF1, PPARG, TRIM5, TRIM22, CRP, AP3B1, ANGPTL3, CHIT1, LGR6, RXFP2, RFXAP, TPT1, KL, NTS, SFTPD, KRAS, SPINK5, TSHB, VAV3, SORT1, NGF, RSAD2, SAA2, IL6ST, GRP, IL2RA, IL15RA, KLRD1, KLRC1, MTNR1B, APLNR, C5AR2, PDGFRA, CTSL, OASL, CGA, AMHR2, LACRT, GDF11, CDK4, ACVR1B, NMBR, TEC, SEMA4F, AGT, RNASEL, STAB2, PLXNC1, EDNRB, RAC1, IL6, IREB2, GH2, CSH1, TANK, IL10, IL36G, IL1RN, IL36A, IL36RN, IL36B, IL1F10, ANGPTL2, TLR4, NR5A1, IL33, IL11RA, CCL21, IFNA21, CD72, BPHL, TLR2, ARRB1, PI15, THBS1, DUOX1, SEMA6D, LHCGR, RBP4, MSTN, ITGAV, SLC40A1, SEMA7A, FGF5, IL21, FGF2, BMPR1B, CXCL9, EGF, PPP3CA, TAPBPL, RBP5, INHBE, LGR5, ACVRL1, NPFF, CELA1, LMBR1L, SSTR1, ESR2, DUOX2, FGF7, IGF1R, PML, FURIN, CMTM3, CMTM2, OSGIN1, PIK3R5, SECTM1, TNFRSF11A, VAV1, IFNAR1, SOD1, AKT1, IL19, CBLC, GPR32, PTH2, IL22RA1, SYTL1, BCL10, TINAGL1, PROK1, RXRG, XCL1, XCL2, NR1I3, ISG20L2, CRABP2, HDGF, RORC, RFX5, SEMA6C, S100A8, S100A7, ACTA1, LEFTY2, GDF7, REG3G, GPR17, PTH2R, ACKR3, ACKR2, IL17RD, NFKBIZ, NR1I2, AGTR1, UCN2, MANF, MUC4, SLIT2, FABP2, PDGFC, OSMR, PIK3R1, TSLP, CXCL14, LECT2, IL9, TNFRSF21, NFKBIE, VIP, EGFR, ZC3HAV1L, IL2RG, GNRH1, CRH, PMP2, CER1, IFNA5, IFNA16, IFNK, SHC3, NR6A1, LCN2, LRSAM1, LCN9, TCF7L2, ADM, PAK1, HTR3B, GPHA2, PPP4C, CTF1, VEGFC, IL18, CRIM1, THRB, EDNRA, NR3C2, CCL28, MCHR2, CYSLTR2, PDK1, PTH, RASGRP3, BMP3, MR1, BMP6, NR4A2, CMTM7, CD8A, SEMA3D, ROBO3, ANGPT1, PRKCA, TMSB4Y, RAET1L, DCK, CXCL13, FGF18, GDF6, NODAL, SST, GHRL, NRG1, IL34, MX1, BRAF, TNFRSF14, NCK1, TMSB15A, COLEC12, TMSB15B, NRG2, CD1D, CD1A, CD1C, CD1B, CD1E, FGF17, FCER1G, IFNAR2, IFNGR2, STC1, CSRP1, GIP, ADIPOR1, PSMD4, UBR1, PGLYRP3, AGRP, ZYX, TNFRSF13C, NR2F6, ITGB2, VAV2, S100B, FCN2, LCN1, TOR2A, CD3G, S100A1, CXCR5, SHC1, NLRX1, ADAR, IL6R, CCR5, PTH1R, AZGP1, FGFR4, PTGER1, PGLYRP2, SCGB3A1, PSMC2, DCD, CXCL16, TNFSF13, FGF11, SSTR5, UBXN1, FGF19, JAK1, RBP7, SDC3, IL23R, GBP2, VCAM1, FCGR3B, IL20, IL24, INHBB, CTSS, S100A11, BMP10, PGLYRP4, S100A9, S100A12, TGFA, NPPC, PROK2, CXCR1, TGFBR2, RETNLB, CTLA4, ICOS, ALB, PSMD6, PTX3, IL17RE, IL17RC, CXCL3, CXCL5, PPBP, PF4, CXCL1, UCN, CCR1, S100P, AIMP1, PGRMC2, CAMP, PLXNB1, IL15, F2RL1, ESM1, CASP3, ERAP1, ERAP2, TLR3, KLKB1, IL3, CSF2, GDF9, LEAP2, FABP7, IL22RA2, IL31RA, RAET1E, FABP5, CTSB, HNF4G, TNFRSF11B, DEFA5, DEFA4, DEFA6, DEFB1, GPER1, SPAG11B, SYK, CYBB, TSHR, MBL2, RNASE7, PSMC3, IL25, CMTM5, AVPR1A, ILK, CRABP1, PRKCB, TK2, MC4R, B2M, HTR3A, CHP2, GREM1, PDIA3, BPIFB6, NOD2, IGF2, CD3D, LALBA, KIR3DL1, SEMA6B, ANGPTL4, CD320, PRDX2, LTBP3, PNOC, PTGDR, CX3CR1, TAP1, MTNR1A, BMP1, STAT3, GDNF, IL7R, SEMA4C, IL12A, INPP5D, MAP2K1, AR, IL13, CXCL10, CXCL11, ADRB2, NR0B1, RNASE2, RNASE3, PTK2, PTAFR, NPR1, CXCL8, SDC2, GKN1, RAC3, NRG4, LIMS1, ROBO1, S100G, FABP6, CMTM8, FABP4, FOS, SEMA3E, CD14, HSPA4, FSHR, TRH, NUDT6, PDGFD, S1PR1, FPR2, FPR1, OBP2B, INSR, NRTN, RLN3, APLN, RXFP1, PTGER4, FGA, PIK3CD, S100Z, DEFB4A, ANGPTL7, IFNB1, C3AR1, SCG2, CD8B, CCL11, MALT1, PRL, ISG20, CXCR6, AZU1, IL16, INSL5, IL17D, RASGRP1, CCL19, RARG, RELA, HSPA6, ESRRA, CYSLTR1, OLR1, SAA1, VEGFB, TNFRSF10D, TNFRSF10C, XCR1, CCR9, PSMD1, TLR1, TRHR, IL20RB, CMKLR1, LEP, NR1D2, HRAS, BTC, DES, PSMD2, INHBC, NPPA, PCSK1, CLCF1, NR2F1, CALCB, A2M, CRLF3, BDNF, DEFB104A, DEFB103A, C8G, DEFB104B, IFNW1, DEFB103B, DEFB4B, UMODL1, CD19, NR2C2, RABEP2, JUN, IL17RA, DEFB114, GRB2, LCN15, HTR3C, SPAG11A, HTR1A, UCN3, AMBN, CD28, DEFB125, CALR, PTPN11, HLA-DQB1, CIITA, GPHB5, CCR8, PAK2, DEFB124, DEFB123, DEFB119, SSTR2, PRF1, AGTR2, CXCR2, DEFB112, GREM2, OXTR, AEN, ADIPOQ, F2R, PENK, CCL13, TNFSF15, IFNL1, CSF1R, EPGN, RXFP3, GALR2, LCK, TCHHL1, PTGDR2, PMCH, MX2, KLRC4, CCR3, BMP8A, IFNL2, CMTM4, TBK1, CCR4, FAM3B, SH2D1A, ACTG1, DEFB108B, S100A7A, GDF3, CSF1, COLEC10, CCR10, BPIFC, GAST, SOCS3, JAG2, LCN12, IFNE, ROBO2, SEMA4B, MC2R, CDNF, IL3RA, SFTPA2, SOCS1, IFNLR1, IRF7, NR2F2, SP1, PDIA2, PSMD13, SHC4, NTF3, NRG3, IFITM1, DEFB128, HTR3E, HTR3D, BPIFB3, BPIFB4, RXRA, DEFB132, DEFB105A, DEFB107A, DEFB106A, DEFB105B, IFNA10, CXCR3, TNFRSF4, MAPT, TNFRSF18, FGF3, PPARA, TMPRSS6, DEFB106B, CCK, SLIT1, ANGPTL5, EPOR, CHP1, ISG15, TMSB4XP8, SEMA4D, DMBT1, LCN10, S100A3, PLA2G2A, PLSCR1, IFNA2, PPP3R2, PDCD1, S100A16, QRFP, OSTN, UTS2B, KIR2DL4, CGB5, APOD, CLDN4, S100A13, S100A14, CXCL17, HMGB1, NCR1, IL1RAP, HLA-DRB1, CTSE, SEMA4A, PPIA, CGB7, PRTN3, S100A5, FGF16, ESRRG, PLXNB2, TLR7, HLA-DQA1, S100A2, FAM3C, ANXA6, GMFB, IGF2R, IFNL3, SRC, IL27, GUCA2A, S100A7L2, C5AR1, FABP12, PDGFA, GALP, ELANE, NMB, S100A10, IFNA1, PLCG2, S100A6, AVPR1B, DEFB107B, BPIFA1, TUBB3, CSF2RA, SLC29A3, CARD11, TPM2, HLA-DRB5, SH2D1B, FAM3D, LPA, PLXNB3, CD247, CD3E, RORB, RAET1G, FCGR3A, DEFB110, LCN8, DAXX, BMPR2, RXRB, HLA-DOA, HLA-DMA, PSMB8, TAP2, HLA-DRA, AGER, SKIV2L, HSPA1B, HSPA1A, HSPA1L, CSHL1, APOM, NCR3, MICB, MICA, HLA-C, DEFB121, LILRB3, HLA-E, HLA-G, HLA-F, IL31, TRIM27, FABP9, LGR4, TMSB4X, HTN3, CRLF2, KLRC2, KLRC3, DEFB134, DEFB135, DEFB136, DEFA7P, DEFA1, HLA-H, HLA-A, IGKC, IGKJ5, IGKJ4, IGKJ3, IGKJ2, IGKJ1, IGKV4-1, IGKV5-2, IGKV6-21, IGKV3D-20, IGKV6D-41, IGKV3D-11, IGKV1D-42, IGLV4-69, IGLV8-61, IGLV4-60, IGLV6-57, IGLV11-55, IGLV10-54, IGLV5-52, IGLV1-51, IGLV1-50, IGLV5-48, IGLV1-47, IGLV7-46, IGLV5-45, IGLV1-44, IGLV7-43, IGLV1-40, IGLV5-37, IGLV1-36, IGLV2-33, IGLV3-32, IGLV3-27, IGLV3-25, IGLV2-23, IGLV3-22, IGLV3-21, IGLV3-19, IGLV2-18, IGLV3-16, IGLV2-14, IGLV3-12, IGLV2-11, IGLV3-10, IGLV3-9, IGLV4-3, IGLV3-1, IGLJ1, IGLC1, IGLJ2, IGLC2, IGLJ3, IGLC3, IGLJ4, IGLJ5, IGLJ6, IGLJ7, IGLC7, TRGJ2, TRGJP2, TRGC1, TRGJ1, TRGJP, TRGJP1, TRGV9, TRGV8, TRGV5, TRGV4, TRGV3, TRBV6-1, TRBV4-1, TRBV6-4, TRBV7-3, TRBV9, TRBV10-1, TRBV11-1, TRBV6-5, TRBV6-6, TRBV5-5, TRBV7-6, TRBV5-6, TRBV5-7, TRBV5-1, TRBV4-2, TRBV19, TRBV20-1, TRBV24-1, TRBV25-1, TRBV27, TRBV28, TRBJ2-1, TRBJ2-2, TRBJ2-3, TRBJ2-4, TRBJ2-5, TRBJ2-6, TRBJ2-7, TRBC2, TRAV2, TRAV3, TRAV4, TRAV5, TRAV6, TRAV7, TRAV8-1, TRAV9-1, TRAV10, TRAV12-1, TRAV8-2, TRAV8-3, TRAV13-1, TRAV12-2, TRAV8-4, TRAV13-2, TRAV14DV4, TRAV9-2, TRAV12-3, TRAV8-6, TRAV16, TRAV17, TRAV18, TRAV19, TRAV20, TRAV21, TRAV22, TRAV23DV6, TRDV1, TRAV24, TRAV25, TRAV26-1, TRAV8-7, TRAV27, TRAV29DV5, TRAV26-2, TRAV34, TRAV35, TRAV36DV7, TRAV38-1, TRAV38-2DV8, TRAV39, TRAV40, TRAV41, TRDV2, TRDJ1, TRDJ4, TRDJ2, TRDJ3, TRDC, TRAJ61, TRAJ59, TRAJ58, TRAJ57, TRAJ56, TRAJ54, TRAJ53, TRAJ52, TRAJ50, TRAJ49, TRAJ48, TRAJ47, TRAJ46, TRAJ45, TRAJ44, TRAJ43, TRAJ42, TRAJ41, TRAJ40, TRAJ39, TRAJ38, TRAJ35, TRAJ34, TRAJ33, TRAJ32, TRAJ31, TRAJ30, TRAJ29, TRAJ28, TRAJ27, TRAJ26, TRAJ25, TRAJ24, TRAJ23, TRAJ22, TRAJ21, TRAJ20, TRAJ19, TRAJ18, TRAJ17, TRAJ16, TRAJ14, TRAJ13, TRAJ12, TRAJ11, TRAJ10, TRAJ9, TRAJ8, TRAJ7, TRAJ6, TRAJ5, TRAJ4, TRAJ3, TRAJ2, TRAJ1, IGHA2, IGHE, IGHG4, IGHG2, IGHA1, IGHG1, IGHG3, IGHD, IGHM, IGHJ6, IGHJ2, IGHJ1, IGHD1-26, IGHD5-24, IGHD3-22, IGHD2-21, IGHD6-19, IGHD5-18, IGHD3-16, IGHD2-15, IGHD6-13, IGHD5-12, IGHD3-10, IGHD3-9, IGHD2-8, IGHD5-5, IGHD3-3, IGHD2-2, IGHV6-1, IGHV1-2, IGHV1-3, IGHV2-5, IGHV3-7, IGHV3-11, IGHV3-13, IGHV3-15, IGHV3-16, IGHV1-18, IGHV3-20, IGHV3-21, IGHV3-23, IGHV1-24, IGHV2-26, IGHV4-28, IGHV3-33, IGHV4-34, IGHV3-35, IGHV3-38, IGHV4-39, IGHV1-45, IGHV1-46, IGHV3-48, IGHV3-49, IGHV5-51, IGHV3-53, IGHV1-58, IGHV4-61, IGHV3-66, IGHV1-69, IGHV2-70, IGHV3-73, IGHV7-81, DEFB117, CGB8, ACKR1, CSH2, NRAS, CHUK, LAT, KLRK1, LTB4R, LTB4R2, CCL27, IRF9, IFNA7, LCNL1, DEFB113, DEFB133, GPR33, MUC5AC, DEFB116, DEFB115, GCGR, TNFRSF25, IFI30, CKLF, MBL3P, PPP3R1, PLXNA4, KIR2DS4, IGLC6, IGLV9-49, IGHV3-64, HLA-DPB1, TRDD1, IGKV3D-15, IGHV4-59, IGHV3-74, TDGF1P3, IGKV6D-21, IGHV3-72, IGHD6-25, NTF4, TRBV2, LTA, IGHD1-14, TRGC2, IGHD4-23, LTB, IGHD4-17, IFNA14, IGHD6-6, ORM2, IGKV3D-7, TRDD3, ORM1, TRBV10-2, TRBV5-4, HLA-DPA1, IGHV4-31, TAPBP, IGHV3-43, IGHD4-11, TNF, TRBV29-1, TRGV2, IGHD4-4, IFNA13, HLA-B, IFNA17, IGHD1-1, IGHD7-27, IFNA4, IGHD1-20, IGHD1-7, TRDD2, TRBV30, HLA-DQA2, TRBV3-1, IGKV2D-30, TNFSF12, APOBEC3G, IGKV1D-8, DEFA3, IGKV1-6, IGKV1-37, IGKV3-20, IGKV1D-33, IGHJ4, DEFA1B, IGKV1-17, KIR3DL2, TNFRSF13B, IGKV1-8, IGKV1-16, MIF, HLA-DOB, TDGF1, IGKV1D-16, IGKV2-24, IGKV3-11, CORT, IGKV2D-24, TRBV11-2, IGKV1-9, KIR3DL3, IGKV1-33, IGKV1-39, IGHJ5, IGKV2D-28, HLA-DMB, IGKV1D-43, CNTF, IGKV1D-17, IGHJ3, IGKV3-7, IGKV2-30, IGKV2D-29, IGKV1-12, IGKV1-5, TNFRSF6B, IL10RB, LEFTY1, KIR2DL3, IGKV2-28, IGKV3-15, APOBEC3C, IGKV1-27, INSL3, PPBPP2, IGKV1D-37, PPBPP1, IGKV2D-40, IGKV1D-39, TRBV6-7, TRBV7-7, TRBV7-4, IGKV1-13, TRBV6-8, LYN, INS, NOX5, TRAV1-1, TRAV1-2, TRDV3, PDF, MC1R, TRAV30, PAK6, GH1, FIGNL2, MIA, MMP12, IKBKE, GDF2, GDF10, LCN6, S1PR2, CGB1, IKBKG, IGHV3-30, CCL5, SERPINA3, IGKV2-40, CCL23, TRBV12-3, CCL16, TRBV12-5, TRBV16, CCL4, CCL18, CCL15-CCL14, CCL15, TRBV14, TRBV10-3, CCL4L1, CCL3L3, TRBV13, CCL14, TRBV18, IGKV1D-13, TRBV11-3, TRAJ36, IGHV4-4, TRBV15, TRBV12-4, CCL3, TRAC, TRBV17, TRBV7-9, IGLV2-8, NR2E3, TRAJ37, IGKV1D-12, IGHV1-69-2* |
| --- |

**Supplementary Table 2: List of genes in each gene co-expression module from WGCNA**

| **Module color** | **Gene symbol** |
| --- | --- |
| Blue | *FGR, PLXND1, CALCR, ITGAL, CX3CL1, TNFRSF12A, CD79B, IL32, GIPR, SEMA3G, CD4, BTK, PLAUR, TYROBP, CD22, SLC11A1, CD74, NR1H3, TYMP, IFNGR1, TNFRSF1B, GRN, FLT4, MSR1, LCP2, CALCRL, TIE1, TNFRSF1A, BCL3, FCGR2B, PLXNA2, IL4R, EDN1, CXCL2, PTPRC, CETP, SH3BP2, OAS1, ICAM1, LYZ, FLT3LG, CD209, CMTM6, BLNK, IL12RB1, NRP1, OSM, HMOX1, PDGFB, CSF2RB, IL2RB, GZMB, LGMN, NFKBIA, PLTP, MMP9, PROCR, CD40, ANGPT4, HCK, JAG1, TLR8, CD40LG, TNFSF13B, FLT1, AQP9, RELB, FCGRT, IL27RA, EBI3, CD79A, CLEC11A, PIK3CG, TNFSF8, ENG, CXCL12, ICAM2, CCL2, CCL8, DHX58, UNC93B1, IL10RA, CD81, VDR, PTPN6, HBEGF, FGF1, CD86, CBLB, IFIH1, LEPR, CD48, NRP2, TNFAIP3, SPP1, CSF3R, LTBP2, TEK, TNFSF11, PTK2B, CCRL2, TNFSF10, CXCR4, PLAU, IRF1, IL1B, C3, HCST, CCR7, IRF3, KDR, RAC2, LIF, IRF5, DLL4, BST2, GMFG, ANGPTL6, NFATC1, NR1H2, IL13RA1, PPARG, TRIM5, TRIM22, RSAD2, IL2RA, IL15RA, APLNR, CTSL, OASL, TEC, RNASEL, EDNRB, TANK, IL10, IL1RN, TLR4, IL33, CD72, TLR2, ARRB1, SLC40A1, SEMA7A, TAPBPL, ACVRL1, LMBR1L, PML, CMTM3, OSGIN1, PIK3R5, SECTM1, TNFRSF11A, VAV1, TINAGL1, S100A8, NFKBIE, IL2RG, VEGFC, IL18, RASGRP3, CMTM7, IL34, MX1, TNFRSF14, FCER1G, IFNAR2, IFNGR2, CSRP1, ZYX, ITGB2, CXCL16, TNFSF13, RBP7, GBP2, VCAM1, FCGR3B, INHBB, CTSS, S100A9, CXCR1, TGFBR2, PTX3, CXCL3, PPBP, PF4, CXCL1, CCR1, ERAP1, CTSB, CYBB, PRKCB, TK2, B2M, NOD2, SEMA6B, CX3CR1, TAP1, IL7R, SEMA4C, INPP5D, CXCL10, RNASE2, PTAFR, NPR1, LIMS1, CMTM8, CD14, S1PR1, FPR2, FPR1, PIK3CD, C3AR1, CYSLTR1, OLR1, TNFRSF10C, TLR1, CMKLR1, IL17RA, CD28, HLA-DQB1, CIITA, CXCR2, F2R, TNFSF15, CSF1R, FAM3B, CSF1, SOCS3, SEMA4B, IL3RA, TNFRSF4, S100A3, PLSCR1, HLA-DRB1, SEMA4A, TLR7, HLA-DQA1, SRC, C5AR1, PDGFA, PLCG2, CSF2RA, SLC29A3, CARD11, HLA-DRB5, FCGR3A, HLA-DOA, HLA-DMA, PSMB8, TAP2, HLA-DRA, HLA-C, LILRB3, HLA-E, HLA-F, TMSB4X, HLA-H, HLA-A, TRGC1, TRGV4, TRBV5-5, IFI30, CKLF, HLA-DPB1, LTB, HLA-DPA1, TAPBP, TNF, HLA-B, HLA-DQA2, TNFSF12, APOBEC3G, HLA-DMB, IL10RB, APOBEC3C, LYN, IKBKE, CCL5, CCL18, CCL4L1, CCL3L3, CCL14, CCL3* |
| Brown | *PSMC4, BID, OGFR, RFXANK, ELAVL1, MYDGF, PSME1, APOBEC3H, GRAP2, PSME2, TRPC4AP, CCL22, PSMD7, RETN, HAMP, IFNG, ZAP70, STAT1, GNLY, FASLG, NENF, INSL6, CD244, TNFSF14, MAP2K2, ADRM1, IDO1, CXCL9, XCL2, LRSAM1, PPP4C, CD8A, CXCL13, NR2F6, TOR2A, CD3G, CCR5, UBXN1, ICOS, CD3D, CXCL11, ADRB2, CD8B, ISG20, CXCR6, AZU1, HRAS, PRF1, LCK, PMCH, MX2, SH2D1A, IRF7, PSMD13, IFITM1, CXCR3, ISG15, PDCD1, IL27, CD247, CD3E, NCR3, TRBV5-1, TRBV4-2, TRBV20-1, TRBV25-1, TRBV28, TRBJ2-3, TRBJ2-4, TRBC2, TRAV2, TRAV14DV4, TRAV19, TRDV1, TRDC, TRAJ31, TRAJ3, IRF9, LTA, TRGC2, TRBV5-4, TRBV29-1, TRGV2, TRBV3-1, IGKV3-15, INSL3, CCL4, TRBV10-3, TRAC, TRBV7-9* |
| Green | *MARCO, NOX4, PTHLH, CXCL6, ACKR4, FGF5, TSLP, CTLA4, F2RL1, CSF2, PTGDR, PTGER4, CLCF1, CCL13, FGF16, IGKV3D-11, TRBV24-1, TRBV27, TRBJ2-1, TRBJ2-2, TRAV3, TRAV36DV7, TRAJ61, IGHD2-8, IGHV4-31, TRBV30, TRAV30, TRBV12-4* |
| Red | *IGKV3D-20, IGLV3-27, IGLV3-19, IGHD3-3, IGHV4-28, IGHV1-46, IGHV4-61, IGHV1-69, IGHV2-70, IGHV4-59, IGKV6D-21, IGKV1-6, IGKV1D-33, IGKV1-17, IGKV1-8, IGKV1-33, IGKV1-39, IGHJ3, IGKV1D-37, IGKV1D-13* |
| Turquoise | *NOX1, PGLYRP1, NR1H4, CDH1, ADRB1, TNFRSF17, PTGER3, GLP2R, FGF22, NCK2, PRLH, NOX3, TXK, FGF4, FGF20, SCTR, OPRK1, DEFB127, CHGB, IL5RA, APOH, ESR1, PGC, NCR2, MLN, DDX17, R3HDML, HNF4A, PDYN, CST4, FGF14, CGB2, LHB, CLEC4M, FGF21, ZC3HAV1, CRHR2, GHRHR, FGF8, CSF3, PPY, VTN, GNRHR, CBL, FGF6, ENDOU, OPRM1, IL17A, IL17F, GLP1R, NR2E1, CCR6, IL12B, IL4, IL5, KNG1, HRG, RBP2, ACVR2B, VIPR1, IL1A, POMC, GCG, TACR1, REG1A, IL1RL1, CR2, MPL, GHRH, LCN1P1, ESRRB, PRLHR, INSL4, IFNA8, NPPB, IAPP, GHSR, FLT3, SFTPA1, MC3R, SEMG2, SEMG1, IL9R, IL17C, SLC10A2, KIR2DL1, IL37, GNRH2, DEFB126, DEFB129, BPIFB1, SLURP1, HTN1, PRKCG, IL22, FGF13, INS-IGF2, BMP15, EPO, BPIFA2, BPIFA3, DEFB118, CCL25, FSHB, NR0B2, CRP, ANGPTL3, CHIT1, TSHB, SAA2, KLRC1, MTNR1B, CGA, AMHR2, LACRT, GH2, IL36G, IL36A, IL36RN, IL1F10, IFNA21, LHCGR, IL21, CELA1, ESR2, DUOX2, IL19, CBLC, GPR32, PROK1, XCL1, NR1I3, RORC, S100A7, REG3G, NR1I2, MUC4, LECT2, IL9, CRH, CER1, IFNA5, IFNA16, IFNK, NR6A1, LCN9, GPHA2, MCHR2, CYSLTR2, PTH, PRKCA, RAET1L, NODAL, SST, NRG1, TMSB15B, CD1B, CD1E, GIP, PGLYRP3, AGRP, S100A1, AZGP1, PGLYRP2, DCD, FGF19, IL20, IL24, PGLYRP4, RETNLB, ALB, IL15, IL3, GDF9, LEAP2, FABP7, IL22RA2, DEFA5, DEFA4, DEFA6, MBL2, IL25, MC4R, HTR3A, CHP2, BPIFB6, LALBA, KIR3DL1, MTNR1A, IL13, GKN1, NRG4, S100G, OBP2B, RXFP1, FGA, S100Z, DEFB4A, CCL11, PRL, INSL5, CCL19, HSPA6, XCR1, TRHR, BTC, NPPA, CALCB, DEFB104A, C8G, IFNW1, DEFB4B, UMODL1, LCN15, HTR3C, SPAG11A, UCN3, DEFB125, GPHB5, DEFB123, DEFB119, DEFB112, IFNL1, EPGN, RXFP3, PTGDR2, CCR3, IFNL2, CCR4, DEFB108B, GDF3, GAST, LCN12, MC2R, SFTPA2, SHC4, DEFB128, HTR3E, HTR3D, BPIFB3, DEFB132, DEFB105A, DEFB106A, IFNA10, FGF3, TMPRSS6, DEFB106B, SLIT1, LCN10, IFNA2, PPP3R2, UTS2B, KIR2DL4, S100A14, CXCL17, CTSE, CGB7, ESRRG, GUCA2A, FABP12, IFNA1, AVPR1B, BPIFA1, SH2D1B, FAM3D, LPA, DEFB110, HSPA1A, CSHL1, DEFB121, IL31, HTN3, CRLF2, DEFB134, DEFB135, DEFB136, IGKJ5, IGKV5-2, IGKV6D-41, IGKV1D-42, IGLV4-60, IGLV11-55, IGLV10-54, IGLV5-45, IGLV5-37, IGLV1-36, IGLV3-22, IGLV2-18, IGLV3-16, IGLV3-12, IGLV4-3, IGLJ2, IGLJ3, IGLJ4, IGLJ6, IGLC7, TRGJ2, TRGJP1, TRGV9, TRBV6-1, TRBV4-1, TRBV6-4, TRBV7-3, TRBV10-1, TRBV11-1, TRBV6-5, TRBV7-6, TRBV5-6, TRBV5-7, TRBV19, TRBJ2-7, TRAV4, TRAV5, TRAV6, TRAV7, TRAV8-1, TRAV9-1, TRAV12-1, TRAV8-2, TRAV13-1, TRAV12-2, TRAV13-2, TRAV9-2, TRAV12-3, TRAV16, TRAV17, TRAV18, TRAV21, TRAV22, TRAV23DV6, TRAV24, TRAV25, TRAV26-1, TRAV8-7, TRAV27, TRAV29DV5, TRAV26-2, TRAV34, TRAV35, TRAV38-1, TRAV40, TRAV41, TRAJ58, TRAJ56, TRAJ52, TRAJ47, TRAJ46, TRAJ45, TRAJ44, TRAJ43, TRAJ41, TRAJ38, TRAJ35, TRAJ33, TRAJ27, TRAJ26, TRAJ25, TRAJ21, TRAJ20, TRAJ19, TRAJ18, TRAJ16, TRAJ14, TRAJ12, TRAJ11, TRAJ10, TRAJ9, TRAJ7, TRAJ6, TRAJ5, TRAJ4, IGHE, IGHJ2, IGHD1-26, IGHD2-21, IGHD3-16, IGHD2-15, IGHD6-13, IGHV3-16, IGHV3-20, IGHV2-26, IGHV3-38, IGHV1-58, IGHV3-73, DEFB117, IFNA7, DEFB133, GPR33, MUC5AC, DEFB116, DEFB115, MBL3P, KIR2DS4, IGLC6, IGHV3-64, TRBV2, IGHD1-14, IFNA14, ORM2, IGKV3D-7, TRBV10-2, IGHV3-43, IGHD4-4, IFNA13, IFNA17, IFNA4, IGHD1-7, TRDD2, IGKV1D-8, IGKV1-37, KIR3DL2, HLA-DOB, TDGF1, IGKV2D-24, KIR3DL3, CNTF, IGKV1D-17, KIR2DL3, IGKV2-28, PPBPP2, IGKV2D-40, TRBV6-7, TRBV7-7, TRBV7-4, IGKV1-13, TRBV6-8, INS, NOX5, TRAV1-1, TRAV1-2, TRDV3, PAK6, GH1, FIGNL2, GDF2, LCN6, CGB1, SERPINA3, IGKV2-40, CCL23, TRBV12-3, CCL16, TRBV12-5, TRBV16, CCL15, TRBV13, TRBV18, TRBV11-3, TRAJ36, TRBV17, NR2E3, TRAJ37* |
| Yellow | *CTF1, IGKC, IGKV4-1, IGKV6-21, IGLV4-69, IGLV8-61, IGLV1-51, IGLV5-48, IGLV1-47, IGLV7-46, IGLV1-44, IGLV7-43, IGLV1-40, IGLV3-25, IGLV2-23, IGLV3-21, IGLV2-14, IGLV2-11, IGLV3-10, IGLV3-9, IGLV3-1, IGLC2, IGLC3, IGHA2, IGHG4, IGHG2, IGHA1, IGHG1, IGHG3, IGHD, IGHM, IGHJ1, IGHV6-1, IGHV1-3, IGHV3-7, IGHV3-11, IGHV3-13, IGHV3-15, IGHV1-18, IGHV3-21, IGHV3-23, IGHV1-24, IGHV3-33, IGHV4-39, IGHV3-48, IGHV3-49, IGHV5-51, IGHV3-53, IGHV3-66, IGLV9-49, IGHV3-74, IGHV3-72, IGKV3-20, IGKV1-16, IGKV1D-16, IGKV2-24, IGKV3-11, IGKV1-9, IGKV2-30, IGKV2D-29, IGKV1-12, IGKV1-5, IGKV1D-39, IGHV3-30, IGLV2-8, IGKV1D-12, IGHV1-69-2* |
| Grey | *NFYA, SEMA3F, MPO, CRLF1, MAP3K14, TAC1, CCL26, ADIPOR2, NOS2, MASP2, HFE, FYN, SEMA3B, LTF, IL20RA, IGF1, HGF, VIM, FAS, RABEP1, TMSB10, TNC, TG, HSPA5, KITLG, UTS2, TNFRSF9, LTBP1, ELN, PIK3CB, KCNH2, EIF2AK2, IL17RB, NGFR, PRKCQ, NFYC, FGFR2, NEO1, RHOA, FGFR3, GAL, RORA, TGFBR3, NEDD4, SCT, FGF10, CDC42, TFRC, NFATC3, PTGS2, EED, SEMA3A, SEMA3C, RARB, PAK3, FGFR1, ARAF, ADCYAP1R1, BPIFB2, HSP90AA1, ARG2, IL12RB2, PGR, SEMA5B, GSK3B, CYLD, TXLNA, PSMC5, MAVS, NOS1, CMTM1, BIRC5, LTBP4, THPO, CMA1, SLC22A17, SEMA6A, TGFB2, PSMD5, SEMA4G, IL11, HSP90AB1, JAK2, PSMD8, SERPIND1, MAPK1, CTSG, SOS2, PSMC6, CHGA, PSMC1, NFATC4, NFATC2, BMP7, AVP, OXT, BPI, WFDC2, EPPIN, PSMD10, MLNR, FGF9, MAPK3, NFAT5, CCL17, IL21R, CSK, PDGFRL, IKBKB, NDRG1, IL7, TNFRSF10A, NFKBIB, AMH, GPI, AKT2, TGFB1, TYK2, PIK3R2, NAMPT, PTN, MET, LMBR1, VIPR2, NOD1, CCL24, TFR2, TGFBR1, C5, OGN, RLN2, RLN1, DDX58, PTGDS, MAPK8, PPP3CB, BMPR1A, MAP3K8, DKK1, PSMD3, PSMD11, CCL7, CCL1, PF4V1, NFKB1, AREG, IL2, HSPA8, MDK, CALCA, IL23A, LTBR, IL26, ULBP1, PPARD, MAPK14, BMP5, BACH2, VEGFA, SEMA5A, GHR, BRD8, ITK, NPR3, PRLR, NR3C1, LIFR, PDGFRB, STC2, RBP1, WNT5A, FGF12, PLXNA1, CSPG5, CCL20, PSMD14, LANCL1, IL1R2, IL1R1, IL1RL2, IL18R1, IL18RAP, PROC, SDC1, SOS1, CACYBP, ANGPTL1, OPRD1, HDAC1, NR5A2, BMP8B, AKT3, ARTN, PRDX1, PIK3R3, TNFSF4, CREB1, FGF23, NR4A3, PGF, TGFB3, CRHR1, IFNA6, TNFSF18, NR2C1, NFYB, TNFRSF10B, PPP3CC, TNFRSF8, CAT, FABP3, PIK3CA, ACVR2A, PAEP, OBP2A, PTGFR, NPY, INHBA, ACO1, CNTFR, ECD, NR4A1, LRP1, IL13RA2, ACVR1C, INHA, PI3, SLPI, SDC4, PLCG1, EDN3, NDP, EREG, AHNAK, ABCC4, AMELX, BMP4, PTGER2, OPRL1, PSPN, TNFSF9, CD70, BMP2, PCSK2, GDF5, THRA, NR1D1, SBDS, BECN1, HSPA2, AVPR2, CANX, EDN2, MASP1, FGFRL1, WFIKKN1, IL17B, TNFRSF19, GNAI1, ADM2, MCHR1, GALR3, APOBEC3A, APOBEC3F, VGF, SHC2, LBP, GDF1, GDF15, JUND, PAK4, PLXNA3, ULBP2, ULBP3, GFAP, PYY, TRAF3, PSME3, RARA, RAF1, AP3B1, LGR6, RXFP2, RFXAP, TPT1, KL, NTS, SFTPD, KRAS, SPINK5, VAV3, SORT1, NGF, IL6ST, GRP, KLRD1, C5AR2, PDGFRA, GDF11, CDK4, ACVR1B, NMBR, SEMA4F, AGT, STAB2, PLXNC1, RAC1, IL6, IREB2, CSH1, IL36B, ANGPTL2, NR5A1, IL11RA, CCL21, BPHL, PI15, THBS1, DUOX1, SEMA6D, RBP4, MSTN, ITGAV, FGF2, BMPR1B, EGF, PPP3CA, RBP5, INHBE, LGR5, NPFF, SSTR1, FGF7, IGF1R, FURIN, CMTM2, IFNAR1, SOD1, AKT1, PTH2, IL22RA1, SYTL1, BCL10, RXRG, ISG20L2, CRABP2, HDGF, RFX5, SEMA6C, ACTA1, LEFTY2, GDF7, GPR17, PTH2R, ACKR3, ACKR2, IL17RD, NFKBIZ, AGTR1, UCN2, MANF, SLIT2, FABP2, PDGFC, OSMR, PIK3R1, CXCL14, TNFRSF21, VIP, EGFR, ZC3HAV1L, GNRH1, PMP2, SHC3, LCN2, TCF7L2, ADM, PAK1, HTR3B, CRIM1, THRB, EDNRA, NR3C2, CCL28, PDK1, BMP3, MR1, BMP6, NR4A2, SEMA3D, ROBO3, ANGPT1, TMSB4Y, DCK, FGF18, GDF6, GHRL, BRAF, NCK1, TMSB15A, COLEC12, NRG2, CD1D, CD1A, CD1C, FGF17, STC1, ADIPOR1, PSMD4, UBR1, TNFRSF13C, VAV2, S100B, FCN2, LCN1, CXCR5, SHC1, NLRX1, ADAR, IL6R, PTH1R, FGFR4, PTGER1, SCGB3A1, PSMC2, FGF11, SSTR5, JAK1, SDC3, IL23R, S100A11, BMP10, S100A12, TGFA, NPPC, PROK2, PSMD6, IL17RE, IL17RC, CXCL5, UCN, S100P, AIMP1, PGRMC2, CAMP, PLXNB1, ESM1, CASP3, ERAP2, TLR3, KLKB1, IL31RA, RAET1E, FABP5, HNF4G, TNFRSF11B, DEFB1, GPER1, SPAG11B, SYK, TSHR, RNASE7, PSMC3, CMTM5, AVPR1A, ILK, CRABP1, GREM1, PDIA3, IGF2, ANGPTL4, CD320, PRDX2, LTBP3, PNOC, BMP1, STAT3, GDNF, IL12A, MAP2K1, AR, NR0B1, RNASE3, PTK2, CXCL8, SDC2, RAC3, ROBO1, FABP6, FABP4, FOS, SEMA3E, HSPA4, FSHR, TRH, NUDT6, PDGFD, INSR, NRTN, RLN3, APLN, ANGPTL7, IFNB1, SCG2, MALT1, IL16, IL17D, RASGRP1, RARG, RELA, ESRRA, SAA1, VEGFB, TNFRSF10D, CCR9, PSMD1, IL20RB, LEP, NR1D2, DES, PSMD2, INHBC, PCSK1, NR2F1, A2M, CRLF3, BDNF, DEFB103A, DEFB104B, DEFB103B, CD19, NR2C2, RABEP2, JUN, DEFB114, GRB2, HTR1A, AMBN, CALR, PTPN11, CCR8, PAK2, DEFB124, SSTR2, AGTR2, GREM2, OXTR, AEN, ADIPOQ, PENK, GALR2, TCHHL1, KLRC4, BMP8A, CMTM4, TBK1, ACTG1, S100A7A, COLEC10, CCR10, BPIFC, JAG2, IFNE, ROBO2, CDNF, SOCS1, IFNLR1, NR2F2, SP1, PDIA2, NTF3, NRG3, BPIFB4, RXRA, DEFB107A, DEFB105B, MAPT, TNFRSF18, PPARA, CCK, ANGPTL5, EPOR, CHP1, TMSB4XP8, SEMA4D, DMBT1, PLA2G2A, S100A16, QRFP, OSTN, CGB5, APOD, CLDN4, S100A13, HMGB1, NCR1, IL1RAP, PPIA, PRTN3, S100A5, PLXNB2, S100A2, FAM3C, ANXA6, GMFB, IGF2R, IFNL3, S100A7L2, GALP, ELANE, NMB, S100A10, S100A6, DEFB107B, TUBB3, TPM2, PLXNB3, RORB, RAET1G, LCN8, DAXX, BMPR2, RXRB, AGER, SKIV2L, HSPA1B, HSPA1L, APOM, MICB, MICA, HLA-G, TRIM27, FABP9, LGR4, KLRC2, KLRC3, DEFA7P, DEFA1, IGKJ4, IGKJ3, IGKJ2, IGKJ1, IGLV6-57, IGLV5-52, IGLV1-50, IGLV2-33, IGLV3-32, IGLJ1, IGLC1, IGLJ5, IGLJ7, TRGJP2, TRGJ1, TRGJP, TRGV8, TRGV5, TRGV3, TRBV9, TRBV6-6, TRBJ2-5, TRBJ2-6, TRAV10, TRAV8-3, TRAV8-4, TRAV8-6, TRAV20, TRAV38-2DV8, TRAV39, TRDV2, TRDJ1, TRDJ4, TRDJ2, TRDJ3, TRAJ59, TRAJ57, TRAJ54, TRAJ53, TRAJ50, TRAJ49, TRAJ48, TRAJ42, TRAJ40, TRAJ39, TRAJ34, TRAJ32, TRAJ30, TRAJ29, TRAJ28, TRAJ24, TRAJ23, TRAJ22, TRAJ17, TRAJ13, TRAJ8, TRAJ2, TRAJ1, IGHJ6, IGHD5-24, IGHD3-22, IGHD6-19, IGHD5-18, IGHD5-12, IGHD3-10, IGHD3-9, IGHD5-5, IGHD2-2, IGHV1-2, IGHV2-5, IGHV4-34, IGHV3-35, IGHV1-45, IGHV7-81, CGB8, ACKR1, CSH2, NRAS, CHUK, LAT, KLRK1, LTB4R, LTB4R2, CCL27, LCNL1, DEFB113, GCGR, TNFRSF25, PPP3R1, PLXNA4, TRDD1, IGKV3D-15, TDGF1P3, IGHD6-25, NTF4, IGHD4-23, IGHD4-17, IGHD6-6, TRDD3, ORM1, IGHD4-11, IGHD1-1, IGHD7-27, IGHD1-20, IGKV2D-30, DEFA3, IGHJ4, DEFA1B, TNFRSF13B, MIF, CORT, TRBV11-2, IGHJ5, IGKV2D-28, IGKV1D-43, IGKV3-7, TNFRSF6B, LEFTY1, IGKV1-27, PPBPP1, PDF, MC1R, MIA, MMP12, GDF10, S1PR2, IKBKG, CCL15-CCL14, TRBV14, IGHV4-4, TRBV15* |

**Supplementary Table 3: Summary of top 20 GO terms (Biological process) that genes within the blue module enriched**

| **ID** | **Description** |
| --- | --- |
| GO:0034341 | response to interferon-gamma |
| GO:0060326 | cell chemotaxis |
| GO:0050900 | leukocyte migration |
| GO:0071346 | cellular response to interferon-gamma |
| GO:0030595 | leukocyte chemotaxis |
| GO:0097529 | myeloid leukocyte migration |
| GO:0060333 | interferon-gamma-mediated signaling pathway |
| GO:0001819 | positive regulation of cytokine production |
| GO:0070661 | leukocyte proliferation |
| GO:0032103 | positive regulation of response to external stimulus |
| GO:0071621 | granulocyte chemotaxis |
| GO:0030593 | neutrophil chemotaxis |
| GO:0097530 | granulocyte migration |
| GO:0070663 | regulation of leukocyte proliferation |
| GO:1990266 | neutrophil migration |
| GO:0045088 | regulation of innate immune response |
| GO:0002237 | response to molecule of bacterial origin |
| GO:0032944 | regulation of mononuclear cell proliferation |
| GO:0032943 | mononuclear cell proliferation |
| GO:0042110 | T cell activation |

**Supplementary Table 4: Result of univariate cox analysis**

| **Gene symbol** | **p.value** | **HR (95% CI for HR)** |
| --- | --- | --- |
| *TNFRSF12A* | 0.0309 | 0.68 (0.48-0.97) |
| *SLC11A1* | 0.0413 | 0.46 (0.22-0.97) |
| *IFNGR1* | 0.00663 | 0.32 (0.14-0.73) |
| *GRN* | 0.0102 | 0.59 (0.39-0.88) |
| *MSR1* | 0.0334 | 0.64 (0.42-0.97) |
| *LCP2* | 0.0263 | 0.53 (0.31-0.93) |
| *TNFRSF1A* | 0.000244 | 0.3 (0.16-0.57) |
| *FCGR2B* | 0.0183 | 0.32 (0.12-0.82) |
| *PTPRC* | 0.0432 | 0.62 (0.39-0.99) |
| *SH3BP2* | 0.0089 | 0.38 (0.18-0.78) |
| *CD209* | 0.0159 | 0.57 (0.37-0.9) |
| *HMOX1* | 0.0322 | 0.74 (0.56-0.97) |
| *LGMN* | 0.0263 | 0.53 (0.31-0.93) |
| *HCK* | 0.0435 | 0.66 (0.44-0.99) |
| *TLR8* | 0.0416 | 0.22 (0.052-0.94) |
| *PIK3CG* | 0.045 | 0.37 (0.14-0.98) |
| *TNFSF8* | 0.00435 | 0.22 (0.079-0.63) |
| *ENG* | 0.0192 | 0.53 (0.31-0.9) |
| *CXCL12* | 0.0114 | 0.63 (0.44-0.9) |
| *CCL2* | 0.00768 | 0.58 (0.39-0.87) |
| *CCL8* | 0.0196 | 0.52 (0.3-0.9) |
| *CSF3R* | 0.0207 | 0.36 (0.15-0.86) |
| *PPARG* | 0.000849 | 0.46 (0.29-0.72) |
| *IL2RA* | 0.00884 | 0.21 (0.068-0.68) |
| *IL10* | 0.0295 | 0.19 (0.041-0.85) |
| *TLR2* | 0.0139 | 0.51 (0.3-0.87) |
| *PML* | 0.00371 | 0.29 (0.12-0.67) |
| *CMTM3* | 0.0257 | 0.52 (0.3-0.92) |
| *PIK3R5* | 0.00872 | 0.32 (0.14-0.75) |
| *VAV1* | 0.0142 | 0.47 (0.26-0.86) |
| *IL2RG* | 0.0377 | 0.62 (0.39-0.97) |
| *CMTM7* | 0.00713 | 0.35 (0.16-0.75) |
| *FCER1G* | 0.0124 | 0.72 (0.56-0.93) |
| *IFNAR2* | 0.0322 | 0.44 (0.21-0.93) |
| *ZYX* | 2.67E-05 | 0.26 (0.14-0.49) |
| *GBP2* | 0.00424 | 0.56 (0.38-0.83) |
| *VCAM1* | 0.00838 | 0.65 (0.47-0.89) |
| *RNASE2* | 0.0366 | 0.49 (0.25-0.96) |
| *CD14* | 0.0475 | 0.77 (0.6-1) |
| *FPR1* | 0.00798 | 0.45 (0.25-0.81) |
| *C3AR1* | 0.00993 | 0.61 (0.42-0.89) |
| *CYSLTR1* | 0.0495 | 0.39 (0.16-1) |
| *TNFRSF10C* | 0.00893 | 0.27 (0.1-0.72) |
| *TLR1* | 0.0497 | 0.46 (0.21-1) |
| *CMKLR1* | 0.0183 | 0.58 (0.36-0.91) |
| *TLR7* | 0.0363 | 0.46 (0.22-0.95) |
| *CARD11* | 0.0323 | 0.35 (0.13-0.91) |
| *LILRB3* | 0.0277 | 0.3 (0.1-0.87) |
| *TMSB4X* | 0.021 | 0.63 (0.42-0.93) |

**Supplementary Table 5: The correlation between risk score and immune checkpoints**

|  | ***CD274*** | ***CTLA4*** | ***TIGIT*** | ***PDCD1*** | ***PDCD1LG2*** | **Riskscore** |
| --- | --- | --- | --- | --- | --- | --- |
| ***CD274*** | 1 | 0.589 | 0.581 | 0.538 | 0.709 | -0.560 |
| ***CTLA4*** | 0.589 | 1 | 0.748 | 0.561 | 0.554 | -0.607 |
| ***TIGIT*** | 0.581 | 0.748 | 1 | 0.534 | 0.536 | -0.581 |
| ***PDCD1*** | 0.538 | 0.561 | 0.534 | 1 | 0.491 | -0.479 |
| ***PDCD1LG2*** | 0.709 | 0.554 | 0.536 | 0.491 | 1 | -0.508 |
| **Riskscore** | -0.560 | -0.607 | -0.581 | -0.479 | -0.508 | 1 |
