# Supplementary figures and images for: The Heterogeneity of Infiltrating Macrophages in Metastatic Osteosarcoma and Its Correlation with Immunotherapy
Source: J Oncol. 2021 Jul 21;2021:4836292. doi: 10.1155/2021/4836292 (PMC8321719; doi:10.1155/2021/4836292)

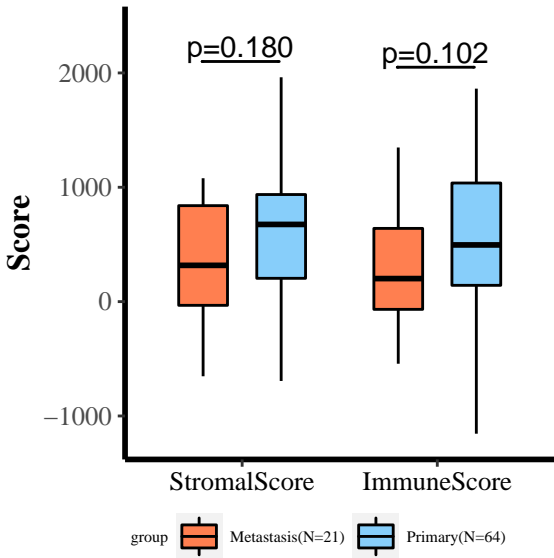

Supplement: Supplementary Materials — Supplementary Figure 1: the stromal score and immune score between primary and metastatic osteosarcoma in TARGET database. Supplementary Figure 2: time-dependent ROC analysis indicating higher predictive value of immune score in OS (left panel, 3 years AUC = 0.702, 5 years AUC = 0.702) and DFS (right panel, 3 years AUC = 0.654, 5 years AUC = 0.644). Abbreviation: ROC : receiver operating characteristic curve. Supplementary Figure 3: the box plot shows the macrophages' infiltration between patients with different metastatic sites (only lung versus bone and lung). Supplementary Figure 4: sample clustering to detect outliers; 85 osteosarcoma patients were in the clusters and 79 patients have passed the cut. [file 4836292.f1.zip › 4836292.f1/Supplementary figure 1.pdf]

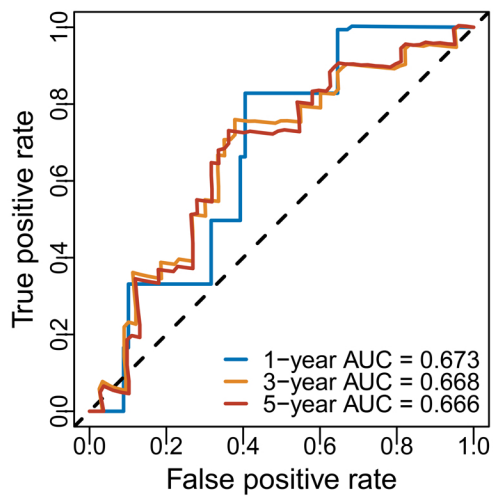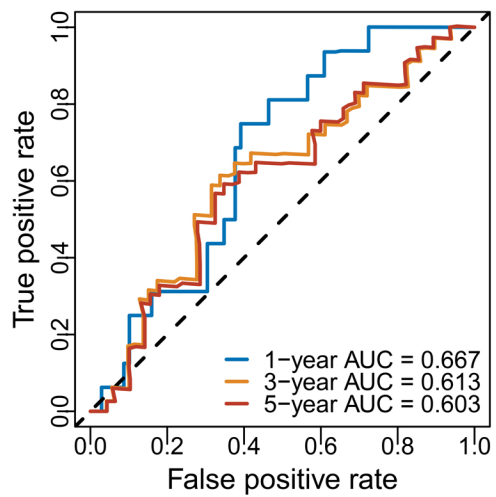

Supplement: Supplementary Materials — Supplementary Figure 1: the stromal score and immune score between primary and metastatic osteosarcoma in TARGET database. Supplementary Figure 2: time-dependent ROC analysis indicating higher predictive value of immune score in OS (left panel, 3 years AUC = 0.702, 5 years AUC = 0.702) and DFS (right panel, 3 years AUC = 0.654, 5 years AUC = 0.644). Abbreviation: ROC : receiver operating characteristic curve. Supplementary Figure 3: the box plot shows the macrophages' infiltration between patients with different metastatic sites (only lung versus bone and lung). Supplementary Figure 4: sample clustering to detect outliers; 85 osteosarcoma patients were in the clusters and 79 patients have passed the cut. [file 4836292.f1.zip › 4836292.f1/Supplementary figure 2.pdf]

Macrophages

Wilcoxon,  $p = 0.45$

0.15

0.10

0.05

0.00

Lung only(N=16)

Bone and lung(N=5)

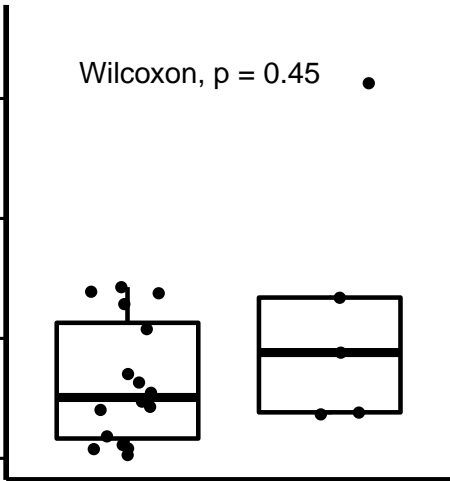

Supplement: Supplementary Materials — Supplementary Figure 1: the stromal score and immune score between primary and metastatic osteosarcoma in TARGET database. Supplementary Figure 2: time-dependent ROC analysis indicating higher predictive value of immune score in OS (left panel, 3 years AUC = 0.702, 5 years AUC = 0.702) and DFS (right panel, 3 years AUC = 0.654, 5 years AUC = 0.644). Abbreviation: ROC : receiver operating characteristic curve. Supplementary Figure 3: the box plot shows the macrophages' infiltration between patients with different metastatic sites (only lung versus bone and lung). Supplementary Figure 4: sample clustering to detect outliers; 85 osteosarcoma patients were in the clusters and 79 patients have passed the cut. [file 4836292.f1.zip › 4836292.f1/Supplementary figure 3.pdf]

# Sample clustering to detect outliers

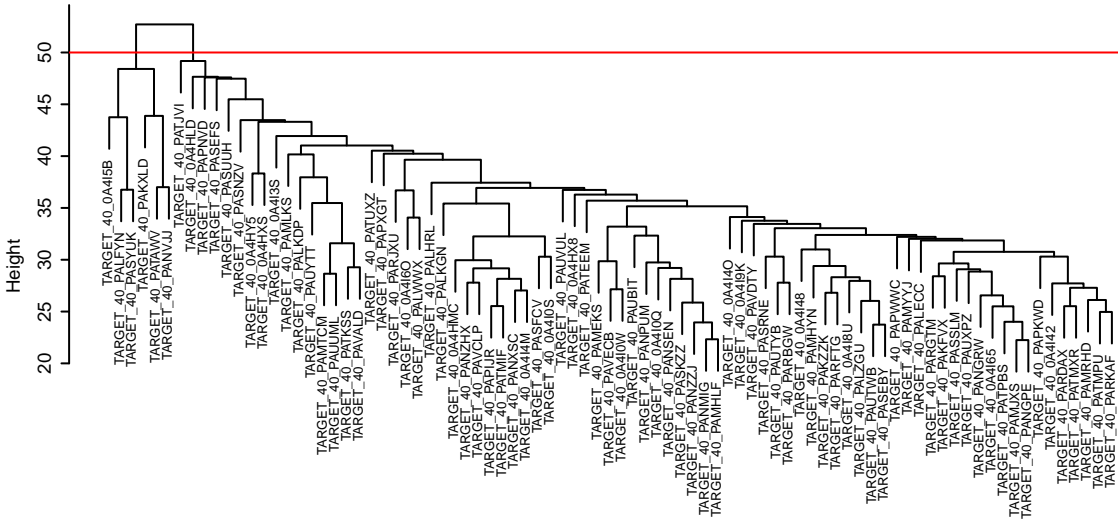

Supplement: Supplementary Materials — Supplementary Figure 1: the stromal score and immune score between primary and metastatic osteosarcoma in TARGET database. Supplementary Figure 2: time-dependent ROC analysis indicating higher predictive value of immune score in OS (left panel, 3 years AUC = 0.702, 5 years AUC = 0.702) and DFS (right panel, 3 years AUC = 0.654, 5 years AUC = 0.644). Abbreviation: ROC : receiver operating characteristic curve. Supplementary Figure 3: the box plot shows the macrophages' infiltration between patients with different metastatic sites (only lung versus bone and lung). Supplementary Figure 4: sample clustering to detect outliers; 85 osteosarcoma patients were in the clusters and 79 patients have passed the cut. [file 4836292.f1.zip › 4836292.f1/Supplementary figure 4.pdf]
